# Elucidating Mental Health Disorders among Rohingya Refugees: A Malaysian Perspective

**DOI:** 10.3390/ijerph17186730

**Published:** 2020-09-15

**Authors:** Kushilpal Kaur, Ahmad Hatim Sulaiman, Chee Kok Yoon, Aili Hanim Hashim, Manveen Kaur, Koh Ong Hui, Zuraida Ahmad Sabki, Benedict Francis, Sarbhan Singh, Jesjeet Singh Gill

**Affiliations:** 1 Department of Psychological Medicine, University of Malaya, Kuala Lumpur 50603, Malaysia; hatim@um.edu.my (A.H.S.); ailihas@um.edu.my (A.H.H.); manveen@um.edu.my (M.K.); ohkoh@um.edu.my (K.O.H.); zuraidasabki@um.edu.my (Z.A.S.); ben.franciscan@gmail.com (B.F.); jesjeet@um.edu.my (J.S.G.); 2 Department of Psychiatry and Mental Health Service, Hospital Kuala Lumpur, Kuala Lumpur 50586, Malaysia; cheekokyoon@yahoo.com; 3 Institute for Medical Research (IMR), Ministry of Health, Kuala Lumpur 50588, Malaysia; lssarbhan@moh.gov.my

**Keywords:** mental health disorders, refugees, Rohingya

## Abstract

Mental health disorders (MHDs) among refugees has been recognized as a major public health issue. However, to date, there is limited evidence on the prevalence of MHDs among Rohingya refugees in Malaysia. This study aimed to examine the prevalence and associated factors of major depressive disorder (MDD), generalized anxiety disorder (GAD), and post-traumatic stress disorder (PTSD) among Rohingya refugees in Malaysia. A total of 220 refugees were randomly selected to participate in this cross-sectional study, conducted from June 2019 to November 2019. Perceived social support, religious orientation, food security, and sociodemographic characteristics were assessed as independent variables. The dependent variables assessed were MDD, GAD, and PTSD. The prevalence of GAD, PTSD, and MDD was reported at 92 (41.8%), 84 (38.2%), and 71 (32.3%). Several factors were significantly associated with MDD following multivariate analysis such as perceived low to moderate social support (AOR = 2.17; 95% CI 1.13, 4.19) and food insecurity (AOR = 2.77; 95% CI 1.19, 6.47). Exposure to violence (AOR = 38.46; 95% CI 16.27, 90.91) and food insecurity (AOR = 3.74; 95% CI 1.41, 9.91) were significantly associated with PTSD. Addressing these risk factors could be key in improving mental health outcomes among this vulnerable population.

## 1. Introduction

Refugees are individuals forced to leave their country of origin or habitual residence and are unable to return home safely [1]. These individuals have a fear of persecution because of their race, religion, nationality, and are unable to avail protection for themselves [1]. According to the United Nations High Commissioner for Refugees (UNHCR) as of June 2020, estimated there are 26 million refugees globally, many of whom originate from Syria, Venezuela, Afghanistan, South Sudan, and Myanmar [2]. In Malaysia, there are some 178,990 refugees in 2019, of which the majority are Rohingya refugees from Myanmar (*n* = 101,010) [2]. In the period from 2012 to 2015, many arrived by boat in Thailand after undertaking dangerous journeys across the Andaman Sea and then were smuggled or trafficked into Malaysia [2]. The Rohingya refugees live throughout Peninsular Malaysia, and are considered illegal or prohibited immigrants [1,2]. While in Malaysia, the Rohingya refugees live in overcrowded houses, have minimal access to educational opportunities, employment, healthcare, and social protection or support [3]. Some Rohingya refugees have lived for decades in Malaysia and established livelihoods or receive remittances from relatives resettled in other countries [3]. However, they continue to live in precarious economic situations [3].

While many Rohingya refugees experience physical ill health following injuries or hunger, far more suffer psychological harm [1]. Therefore, there is a growing concern about the mental health consequences of these vulnerable populations. Previous studies have reported that major depressive disorder (MDD), generalized anxiety disorder (GAD), and post-traumatic stress disorder (PTSD) are the most prevalent mental health disorders (MHDs) affecting refugees worldwide [4]. Globally, the estimated prevalence of MDD, GAD, and PTSD among refugees was reported at 44%, 40%, and 36% [4,5]. The evidence on the prevalence of MHDs among Rohingya refugees specifically is very limited and majority were focused on Rohingya refugees in Bangladesh, whereby the prevalence of MDD, PTSD, and GAD was reported at 89%, 36%, and 14% [6]. Shaw et al. (2018) reported a much higher prevalence of 98.8% MDD, GAD, and PTSD among refugees in Malaysia. Several studies have identified several risk factors for MHDs among refugees globally such as poor educational levels, unemployment, unmarried, exposure to violence and physical injuries, poor social support, poor religious orientation, and food insecurity [7,8,9]. Aside from a study conducted among Rohingya refugees in Bangladesh that reported being female and older age as significant risk factors for MDD and PTSD [6], there is still a paucity of studies that have reported on the factors associated with MHDs among Rohingya refugees worldwide. Furthermore, the majority of studies on MHDs among refugees were conducted in high-income countries and non-conflict countries while limited research has been done in first transit areas such as Malaysia and Thailand [4,9].

In Malaysia to date, the majority of the studies examining the prevalence and associated factors of MHDs among Rohingya refugees were conducted among adolescents [9,10], utilized nondiagnostic based mental health screening instruments [11,12], or did not report on the association with perceived social support, religious orientation, food insecurity and MHDs [11,12]. There are also unpublished reports conducted by non-governmental organizations (NGOs) [8]. Therefore, more studies examining the prevalence and associated factors of MHDs among Rohingya refugees in Malaysia are needed as they would highlight the burden of MHDs among this vulnerable population, which otherwise would never surface. Additionally, such studies as this one would provide some understanding regarding the sociodemographic profile and factors (risk and protective) for MHDs among the Rohingya refugees. Furthermore, as Malaysia is a transit country for refugees, most of these refugees are under constant conflict and they require periodic mental health assessment. Unfortunately, they face difficulties accessing basic universal healthcare [1,11]. Current programs that look into the health and welfare of these refugees are mainly conducted by UNHCR, Malaysia, and some NGOs [12]. Therefore, generating more evidence-informed policies for this underprivileged population would require more local based studies.

With the rising number of Rohingya refugees in Malaysia (from 73,900 in 2018 to 101,010 in 2019) [2], these individuals continue to live in harsh living conditions, and with the lack of healthcare support, it is for sure that many have undetected mental health difficulties. Therefore, this study aimed to determine the prevalence of MDD, GAD, and PTSD among adult Rohingya refugees in Malaysia. Besides, this study also examined factors (such as perceived social support, religious orientation, food insecurity, educational attainment, employment status, duration of displacement, homelessness, exposure to violence, and physical injuries) associated with MDD, GAD, and PTSD.

## 2. Materials and Methods

### 2.1. Study Design and Setting

The cross-sectional study design was selected to answer the objectives of this study because it is practical in obtaining baseline data. The study population includes adult Rohingya refugees ages 18 years and above, which ages were verified using the date of birth stated in their UNHCR identity cards, and who provided consent to participate in this study. Exclusion criteria include non-Rohingya refugees. Selangor is one of the states located in Peninsular Malaysia, which reported the highest number of Rohingya refugees nationwide. There are an estimated 66,030 UNHCR persons of concern in Selangor, which includes Rohingya refugees, therefore making Selangor the state with the highest number of refugees in Malaysia, accounting for almost 50% of the total refugee population and more specifically 75% of the total Rohingya refugees [2,11].

### 2.2. Sampling Strategy

A random sampling of all Rohingya refugees residing in Selangor was performed. The registry with names and contact numbers of Rohingya refugees residing in Selangor was obtained from the Rohingya Society of Malaysia (RSM). Random selection was performed using an Excel RAND function. A list of 11,000 randomly generated numbers was obtained from the Excel RAND formula, then the random values generated were ordered and the first 220 subjects in the sorted list were selected. Following which the researcher contacted the selected 220 subjects in the registry (with help from the RSM Office Secretary) to set an appointment to meet the subjects at their residence to further explain the study and obtain written consent.

### 2.3. Sample Size Calculation

A minimum sample of 220 was required after 5% inflation with the power and alpha set at 80% and 0.05 respectively. The G* power version 3 sample size calculator was used to estimate the sample size [13]. Parameters sourced from a study by Feyera et al. (2015) that examined factors associated with MDD amongst adult refugees was used to populate the G* power software, such as the (a) odds ratio (adjusted for the effect of gender) = 2.4, (b) the probability of an outcome (MDD) among participants without exposure (male gender) = 0.26, and (c) the proportion of those with the outcome (MDD) who have the exposure (female gender) = 0.65 as shown in Appendix A [14,15].

### 2.4. Data Collection

Data collection was conducted from July 2019 to August 2019. Training of translators was conducted by the researcher, to inform the purpose of the study and requirements to answer the questionnaire and obtain written consent from participants. Translators were engaged during the process of data collection. Translators were recruited from the RSM and they are well versed with Rohingya, Malay, and the English language. The translators were given training sessions, whereby during this session all the translators went through the items in the subject information sheet, consent forms, and study instruments (questionnaires) that were used in this study so that they were familiar with the meaning of each item. In the event, certain terminologies in the questionnaire’s subject information sheet and consent forms have no direct translation into the Rohingya language, the closest meaning of the words in the Rohingya language was obtained from the glossary report by Tay et al. (2018) [8]. The translators were only used in the event and participants were unable to understand items in the subject information sheet, consent forms, and study instruments. Questionnaires were distributed to all participants who complied with the inclusion criteria during the designated dates for the questionnaire session. The timing of collection and answering the questionnaires was based upon the allocated time agreed upon participants so to ensure that the research process does not interfere with the daily duties of participants. Participants filled in the questionnaire in their residence, which took approximately 30 min to complete. The researcher was present during each questionnaire session to ensure independent responding and also to address any inquiries from participants relating to the questionnaire. Subsequently, the researcher conducted the MINI International Neuropsychiatric Interview on participants that have completed the self-administered questionnaires. A total of 220 questionnaires were collected during the process of data collection.

### 2.5. Measures

Perceived social support using the multidimensional scale of perceived social support (MSPSS) is a 12 item self-administered measure of social support that assesses the perception of social support adequacy from three different sources namely family, friends, and significant others [16]. The items are scored on a 7-point Likert scale (1 very strongly disagree, 7 very strongly agree). The classification of perceived social support is based on the mean scale scores as the following: (a) low to moderate social support (score of 1–5) and (b) high social support (score of 5.1–7) [16]. The MSPSS has been translated to Malay and is reported to have satisfactory psychometric properties for use among adults in Malaysia [17]. In this study, the internal consistency Cronbach alpha was reported at 0.91.

Religious orientation scale-revised (ROS-R) is a 14 item self-administered measure of religious orientations that assess three important dimensions of religious orientations namely intrinsic, socially orientated-extrinsic, and personally orientated-extrinsic [18]. All the items in ROS-R were rated on 5-point Likert scales ranging from strongly disagree (1) to strongly agree (5). Resulting in a range of 8–40 for the intrinsic orientation scale and 3–15 for each extrinsic orientation scale. Higher scores indicate higher levels of a specific religious orientation. The ROS-R has been translated to Malay and reported to have satisfactory psychometric properties for use among adults in Malaysia [19]. In this study, the internal consistency Cronbach alpha was reported at 0.71.

The Radimer/Cornell food insecurity instrument is a 10 item self-administered measure of food security, household food insecurity, individual food insecurity, and child hunger [20]. All the items in this instrument were rated on 3-point Likert scales ranging from 1 (not true), 2 (sometimes true), to 3 (often true). The response is then categorized into negative answers (response not true) and positive answers (response sometimes true or often true). Subsequently, for each measure of food security, the following classification was used: (a) food security is considered when negative answers are scored across all hunger and food insecurity items, (b) household insecure is considered when positive answers are scored to one or more items (1–4) but not to adult or child level items, (c) individual insecure is classified when positive answers are scored to one or more of items (5–8) but not to items (9–10), and (d) child hunger is considered when a positive answer is scored to items (9–10) [21]. The food security status is collapsed into two categories, household food security and household food insecurity, for analysis for this study. The Radimer/Cornell food insecurity instrument has been translated to Malay and reported to have satisfactory psychometric properties for use among the adult population in Malaysia [22]. In this study, the internal consistency Cronbach alpha was reported at 0.95.

The MINI International Neuropsychiatric Interview version 7.02 was designed as a brief structured interview for the major psychiatric disorders in DSM-5 and ICD-10 [23]. Validation and reliability studies have been done comparing the MINI to the SCID-P for DSM-III-R and the CIDI (a structured interview developed by the World Health Organization) [23]. The results of these studies show that the MINI has similar reliability and validity properties, but can be administered in a much shorter period (mean 18.7 ± 11.6 min, median 15 min) than the above-referenced instruments. The ratting responses are either ‘Yes’ or ‘No’. For this study, the MINI was used to assess MDD, PTSD, and GAD. There are 6, 7, and 4 items in the MDD, PTSD, and GAD modules.

Several factors associated with MHDs from previous studies [6,7,8,9] were measured under the sociodemographic factors such as gender, age, marital status, educational attainment, employment status, marital status, time spent in Malaysia, homelessness, separation from family members, exposure to violence, and physical injuries.

### 2.6. Analytic Approach

Statistical analysis was performed using the Statistical Package for the Social Sciences (SPSS) version 24.0 with a *p*-value of less than 0.05 considered as statistically significant [24]. Descriptive statistics used to characterize the participants were presented in categorical and continuous variables, and reported in percentages and mean where appropriate. To determine the association between the factors (perceived social support, religious orientation, food security, and other covariates) and MHDs (MDD, GAD, and PTSD), multivariate binary logistic regression analysis was performed. The analysis was performed separately for each outcome variable (MDD, GAD, and PTSD). Multicollinearity between categorical variables was examined using the Cramer’s V whereby any two variables with a Cramer’s V value of >0.3 are considered to be strongly correlated [15]. Variance inflation factor (VIF) was used to examine multicollinearity between variables measured on a continuous scale, whereby a VIF value greater than 5 indicates multicollinearity [15]. Interaction among variables was performed using the test of the interaction function in SPSS, whereby a significant value (*p* < 0.05) indicates that an interaction is present [15]. Variables with *p* < 0.25 of a univariate analysis, with no evidence of interaction and multicollinearity, were considered significant and added to the multivariable analysis [15].

### 2.7. Ethics

This study was approved by the University of Malaya Research Ethical Committee (UMREC; reference number: UM. TNC2/UMREC–581). All participants were thoroughly briefed about the study and their written consent was obtained before data collection. Participants found to have mild MDD, PTSD, or GAD were given counseling by the researcher (who is a medical doctor specializing in psychological medicine). In cases of moderate MDD, PTSD, or GAD, they were referred to the clinical psychologist arranged by UNHCR, Malaysia for further management. Those participants with severe MDD, PTSD, or GAD who are unable to afford the financial cost of treatment were referred to UNHCR, Malaysia for further assistance and support; while those who can afford the financial cost of treatment, these participants were referred to the nearest government based psychiatry clinic by the researcher.

## 3. Results

The response rate for the study was 100%. There were 116 (52.7%) male and 104 (47.3%) female participants (Table 1). The mean age of participants was 33.5 years, and the majority of participants were young adults (*n* = 151, 68.6%) between the ages of 18–35 years. The majority of participants were married 176 (80%) and had attained secondary school education (*n* = 119, 54.1%). Most participants lived in Malaysia for more than 3 years (*n* = 187, 85.0%). Slightly more than half of the participants worked part-time or were unemployed 124 (56.4%). Three quarters of the participants were separated from their family members 160 (72.7%) and fortunately, the majority of participants have not been exposed to violence (*n* = 125, 56.8%) and non-accidental physical injuries 157 (71.4%). Slightly more than half of the participants reported having high perceived social support (*n* = 129, 58.6%). Food insecurity was reported by the majority of participants 156 (70.9%). The mean overall religious orientation score was reported at 51.8. The prevalence of GAD, PTSD, and MDD was reported at 92 (41.8%), 84 (38.2%), and 71 (32.3%) as shown in Table 2.

Testing for multicollinearity (Appendix B) among the independent variables significant at 0.25 in the univariate analysis examining the factors associated with MDD, revealed that there were four variables in two pairs that had evidence of multicollinearity as follows: (a) exposure to violence and physical injury (Cramer’s V = 0.463) and (b) gender and employment status (Cramer’s V = 0.631). Exposure to violence and employment status were excluded from multivariate analysis due to the presence of multicollinearity. Similarly, there was evidence of multicollinearity among the independent variables significant at 0.25 in the univariate analysis examining the factors associated with PTSD as follows: (a) gender and separation from a family member (Cramer’s V = 0.401) and (b) exposure to violence and physical injury (Cramer’s V = 0.463). Separation from a family member and physical injury were excluded from the multivariate analysis due to the presence of multicollinearity. There was no significant interaction present between the independent variables and the outcome variable (*p*-value > 0.05). The multivariate analysis factors showed (Table 3) being female (AOR = 2.54; 95% CI 1.21, 5.34), living in Malaysia for less than 1 year (AOR = 6.95; 95% CI 1.89, 25.57), having been physically injured (AOR = 3.25; 95% CI 1.52, 6.96), perceived low to moderate social support (AOR = 2.17; 95% CI 1.13, 4.19), and food insecurity (AOR = 2.77; 95% CI 1.19, 6.47) were significantly associated with MDD. There were no variables significantly associated with GAD following the multivariate analysis as shown in Table 4. Similarly, following the multivariate analysis exposure to violence (AOR = 38.46; 95% CI 16.27, 90.91) and food insecurity (AOR = 3.74; 95% CI 1.41, 9.91) were significantly associated with PTSD (Table 5).

## 4. Discussion

The Rohingya of Myanmar are a severely persecuted minority and represent one of the largest groups of stateless people [6]. Thousands of them reside in refugee camps in South Eastern Bangladesh [6]. In Malaysia the community has been living invisibly for more than three decades, as they have not gained legal status. The cross-sectional study among adult Rohingya refugees’ ages 18 years and above revealed that the prevalence of MDD in this study was 32.3%. The finding was similar to the prevalence of MDD reported among adult refugees in the United States of America (USA; 32%) and Italy (31%) [25,26] but much lower compared to studies among refugees in Malaysia, Australia, and Israel, which reported the prevalence of MDD ranging from 79% to 98% [5,27,28]. Among the studies that reported a similar prevalence of MDD as our study, there were several similarities namely the use of identical instruments to assess for MDD, and the majority of participants were displaced for more than three years [25,26]. In contrast, studies reporting a higher prevalence of MDD vary primarily due to differences in methodology, mainly with the instruments used to examine MDD and variation in the participant’s characteristics such as gender and nationalities. Besides, a higher prevalence of MDD was reported in studies that had recruited participants who were in detention [27]. Hostile conditions in detention centers certainly increase the risk of MDD [27]. To date, there is limited evidence on the prevalence of MDD among Rohingya refugees [8]. Riley et al. (2017) reported that the prevalence of MDD among adult Rohingya refugees in Bangladesh was reported at 89%, which is much higher than this study. Aside from methodological variations, it could be possible that unlike Bangladesh, Malaysia is a middle-income country that has a more robust economy and healthcare system; therefore, this might justify a lower prevalence of MDD among Rohingya refugees in our study.

Our study reported the prevalence of GAD at 41.8%, a finding which is consistent with a systematic review that reported an average pool prevalence of GAD among refugees at 40% [29]. The reasons for this similar finding could be mediated by the fact that the majority of these studies were conducted in middle-income countries. A much higher prevalence of GAD was reported among refugees in Cambodia, USA, and Mexico, which reported a prevalence of GAD at 88%, 77%, and 57% [28,30,31]. These variations could be because, unlike studies conducted among refugees in the USA, Mexico, and Cambodia, the majority of refugees in this study had no exposure to violence. Therefore levels of psychological distress would be much lower [32]. Additionally, a lack of specificity with regards to the type of GAD assessed could also result in different findings. For example, we reported on GAD, while the studies conducted in Cambodia and the USA reported on unspecified anxiety disorders [28,30]. The prevalence of GAD is higher in this study when compared to a study conducted among Rohingya refugees in Bangladesh, which reported a prevalence of GAD at 14% [6]. Once again the methodological variation (instruments and sample size) might have most probably resulted in these differences.

The prevalence of PTSD in this study was 38.2%, which was similar to the prevalence of PTSD reported among refugees in a systematic review, which reported an average pool prevalence of PTSD at 36% [29]. Furthermore, studies in Bangladesh, USA, Africa, and Algeria have all reported a similar prevalence of PTSD among the refugee population, ranging from 36% to 38% [6,33,34]. In contrast, studies in the United Kingdom and Australia reports a much higher prevalence of PTSD, at 76% and 70% [27,35]. While studies involving refugees in Thailand, Ethiopia, and Uganda all reported a much lower prevalence of PTSD ranging from 11.8% to 20% [34,36,37]. The similarity in findings across studies could be due to the similar type of refugee population assessed, as is the case of this study and that in Bangladesh, which both focused on Rohingya refugees. Then, the degree, duration, and the number of traumatic events may contribute to the variation in PTSD prevalence rates across studies [37]. At a higher degree, more recent and frequent exposure to traumatic events would result in a higher prevalence of PTSD.

Aside from methodological variations, it is essential to note that variations in the prevalence of mental health disorders across countries could be mediated by several concepts, theories, and models. For example, based on the healthy migrant effect, refugees resettled in countries with better healthcare systems tend to have lower psychological problems [38]. Better healthcare system results in the more efficient practice of health promotion and prevention activities that would improve the health of refugees. Additionally, based on the migration phase model, refugees residing in transit countries tend to display a higher prevalence of psychological problems compared to those refugees who have been resettled [39], as evident in this study. Refugees residing in transit countries often lack fundamental human rights and privileges, therefore, predisposing them to repeated or prolonged chronic stress, which in turn increase the risk of developing mental health problems. Furthermore, Rohingya refugees are individuals that were forced to migrate from their country of origin and are subjected to prolonged displacement [39]. This factor increases the risk of developing mental disorder compared to migrants.

Our study found among the Rohingya refugees that being female increased the odds of developing MDD among the Rohingya refugees. Similar results have been reported in studies involving Rohingya refugees in Bangladesh [6], Syrian refugees in Iraq [38], Afghan refugees in the Netherlands [39], and Bosnian refugees in Sweden [40]. MDD has always been more common among females for various reasons, such as those attributed to genetics, biological changes associated with puberty, cognitive predisposition, sociocultural factors, and feminine roles or stereotypes [41,42]. This study also found that refugees that have been physically injured had increased odds of developing MDD. This is an important finding as refugees are a group of individuals who are constantly at risk of being physically injured compared to the general population. This finding is consistent with previous studies done involving Syrian refugees in Iraq [38], Afghan refugees in the Netherlands [39], and Vietnamese refugees in Australia [43]. Several mechanisms can mediate this finding. Physical trauma causes both physical and psychological impairment that may result in a reduction in health-related quality of life, which could increase the chances of MDD [44]. In contrast to our findings, a study conducted among Rohingya refugees in Bangladesh reported no significant association between physical trauma and MDD [6]. This discrepancy could be due to methodological variations, namely different instruments used to examine physical injuries, variation in analysis, number of variables controlled for, and sample size.

In this study, participants living in Malaysia for less than a year were more likely to be depressed. This finding implies that newly arrived Rohingya refugees in Malaysia tend to have an increased chance of developing MDD. This is an important finding as any refugee arriving in a new country faces many challenges and obstacles, which is augmented, particularly during their initial arrival period. Previous research points to the potential mental health vulnerability of newly arrived refugees such as in the Sudanese, Iran, Afghan, Indian, and Burmese refugees in Australia [45,46,47,48]. Newly arriving refugees must learn to navigate an entirely new community, language, and cultural system, while simultaneously coping with the loss of their homeland, family and employment challenges, and difficulties in accessing health and social services [48,49]. In our study, the majority of the new arrival refugees were unemployed and unmarried compared to peers with a more extended stay. Therefore, they faced financial uncertainties and poor social support. Lacking necessities, restricted movement, and continued concerns for safety are profound during the early stages [6]. Moreover, as Malaysia is not a party to the 1951 Refugee Convention and neither a resettlement country [1]. There is no legislative and administrative framework in place to protect the refugees [1]. These refugees endure a constant feeling of uncertainty. However, the UNHCR, Malaysia do play an essential role in providing identity documents to these refugees, which would prevent their arrest and detention, including securing their release where necessary [2]. These factors may result in adjustment issues that could increase the odds of MDD among newly arrived refugees. Dissimilar to our findings, previous studies reported poor mental health outcomes among Cambodian, Vietnamese, African, and Sudanese refugees with a longer duration of displacement [50,51,52,53]. This variation could be due to differences in the type (nationality) of the refugee population, whereby variation in cultural practices and beliefs across the various refugee populations exist. The differences in cultural practices and beliefs play a pertinent role in the refugees assimilating or adapting to a new setting, culture, and environment.

We also found that refugees with low to moderate perceived social support had increased the odds of MDD. This is an extremely significant finding as refugees, unlike the general population, are deprived of social support due to their underlying circumstances. Similar findings were noted among Sudanese refugees in Australia [45] and Syrian refugees in Germany and Turkey [54,55]. Several reasons justify this finding. As for human beings, social support, be it from immediate or extended families, friends, and social groups, are important elements ensuring good mental health. Poor social support among refugees deprives them of emotional, informational, tangible, and intangible forms of support. The unavailability of any support creates a sense of isolation while in exile, which increases the chances of depression [45]. Refugees with poor or moderate social support often lack guidance and reliable alliance, which is important for their well-being during their stressful periods [54]. Based on the stress-mobilizing hypothesis, stress encourages individuals to seek social support, but in the case of refugees when the social support is unavailable, this would result in worsening of their stress, which further predisposes them to develop depression [56].

In this study, MDD was also found to be significantly associated with food insecurity. This finding suggests that a lack of access and the inability to maintain food supply increases the odds of MDD. It is not surprising as refugees constantly face issues with food availability. Their food availability is closely related to their poor employment status, lack of social support, and their overall status as an underprivileged population. Similar findings have been reported by previous studies conducted among refugees in South Africa, Sri Lanka, and Canada [57,58,59]. Several mechanisms can mediate this finding. For example, a nutritional deficiency that occurs as a result of food deprivation, consumption of cheaper food, which could be lacking in nutritional value, reduction in food intake to provide more food to other household members could all increase the chances of developing MDD [60]. This is because nutritional deficiencies such as essential fatty acids, folate, and vitamin B12 affect normal brain functioning such as enzymatic activity, cellular and oxidative processes, receptor function, maintenance of neuronal tissue, and synthesis, which have been implicated in the pathogenesis of MDD [60]. Furthermore, individuals who fail to secure food for themselves or their families would experience psychological distress and insecurity, which would predispose them to develop MDD [61].

Regarding the factors associated with PTSD, we found that exposure to violence significantly increases the odds of PTSD among Rohingya refugees. Refugees are frequently exposed to violence as a result of torture, rape, murder, genocide, political violence, and war experience [6]. This is an important finding, as exposure to violence in this already vulnerable population would further predispose them to develop PTSD. Similar results have been replicated in studies involving Syrian refugees in Iraq, Afghan refugees in the Netherlands, and Vietnamese and Bosnian refugees in Australia [38,39,43,62]. Fundamentally PTSD commonly occurs following exposure to a violent terrifying event, this is because violence disrupts several domains namely personal safety, interpersonal attachments, sense of justice, identity, and existential-meaning, which results in various psychosocial responses within these domains [63]. Moreover, studies also suggest that exposure to violence causes disturbances in the left hemispheric function, hyperadrenergic activity in the central nervous system (CNS), stimulation of the serotonergic receptors signaling in the CNS, increase in the corticotrophin-releasing factor in the cerebrospinal fluid, and signaling of the dynorphin/κ opioid receptor in the brain, which all may increase the risk of PTSD [64,65]. Furthermore, in this study majority of the participants were separated from a family member during the process of migration. The experience of being alone may also result in a traumatic experience that could increase the odds of PTSD.

Our study demonstrated that refugees with food insecurity had increased odds of having PTSD. This is an important finding as in this study the majority of refugees suffer from food insecurity, a finding that might be common to many refugees worldwide. Similar findings have been reported by studies conducted among refugees in Uganda [37]. Several mechanisms mediate these findings. Food insecurity results in high-stress levels that are generated through the following ways; first insufficient quantity, quality, or diversity of available foods, second feelings of deprivation, or restricted choice about the amount or type of available foods and third having to engage in procurement strategies such as begging, dependence on charity, stealing, or exchanging sex to obtain food [65]. Furthermore, food insecurity also causes hunger and energy depletion, which affects emotion, cognition, behaviors, and is linked to the recollection of violence and trauma that results in increased chances of PTSD [65]. Repeated prolonged exposure to food insecurity has been conceptualized as a traumatic experience that could elevate the risk of developing symptoms of PTSD in response [65,66,67].

Our findings have to be interpreted in light of several limitations. First, the use of a self-reported questionnaire could lead to information bias as a result of social desirability bias. We tried to minimize this by ensuring the participants that there were no personal identifiers on the questionnaire as well as that confidentiality was maintained. Second, participants in this study were residing in Selangor, and therefore generalizing the findings to refugees residing in other states in Malaysia must be done with caution. However, as Selangor is the state with the highest number of Rohingya refugees in Malaysia, it makes perfect sense to initiate such a study as a starting point. Third, as this study used a cross-sectional design, we were unable to establish temporal relationships. Finally, the presence of multicollinearity among the independent variables resulted in the removal of some variables from the multivariate analysis, therefore the effects of the removed variables on the outcome could not be observed in this study. Future development of a composite variable to represent the variables with evidence of multicollinearity such as the variable being physically injured and exposed to violence should be attempted to overcome issues of multicollinearity.

To the best of our knowledge, this study is the first study that examines factors namely perceived social support, food security, religious orientation, and its association with MDD, GAD, and PTSD among adult Rohingya refugees in Malaysia. The novelty of this pioneering study adds valuable information on the burden of mental health disorder and estimating the degree of perceived social support, religious orientation, and food insecurity among the Rohingya refugees in Malaysia. Other strengths include the use of MINI, which is based on DSM V criteria, performed through face to face interviews undertaken by trained clinicians, and would, therefore, more likely provide the realistic prevalence of MDD, GAD, and PTSD as compared to using self-administered screening tools that may produce speculative results [37].

## 5. Conclusions

The most common MHDs affecting Rohingya refuges were GAD, followed by PTSD and MDD. Factors such as low social support, food insecurity, exposure to violence, and duration since displacement were found to be risk factors of developing MHDs among this population. More specifically food insecurity was a common risk factor for both MDD and PTSD among refugees. This study recommends that mental health education and screening programs for refugees in Malaysia needs to be instituted as soon as possible. These programs would increase mental health literacy especially in terms of identifying risk and protective factors for mental disorders and also enable early detection of MHDs among these underprivileged populations. Besides, more mental health capacity building programs that focus on sensitizing healthcare workers at health clinics and NGOs’ workers regarding the common MHDs, risk, and protective factors of MHDs among refugees are needed. These measures would benefit refugees, as they would be able to receive psychological support early on that would alleviate their symptoms before actually getting specialized help.

Additionally, more efforts are needed to address the issue of violence, food insecurity, and low social support among the Rohingya refugees in Malaysia as these factors were found to increase the odds of MHDs significantly. Such efforts would include developing policies that look into the basic rights to employment and universal health coverage (UHC) for refugees in Malaysia. Additionally, more longitudinal studies examining MHDs among Rohingya refugees in Malaysia are needed to increase comparability and generalization of local research findings and to quantify the burden of MHDs among Rohingya, which would subsequently create an opportunity for more evidence-based policies that would benefit refugees in Malaysia.

## Figures and Tables

**Table 1:** Characteristic of participants (*n* = 220).

Characteristics		Frequency *n* (%)
Gender	Male	116 (52.7)
	Female	104 (47.3)
Age (Years)	18–35	151 (68.6)
	36–55	51 (23.2)
	≥56	18 (8.2)
Marital status	Single	39 (17.7)
	Married	176 (80.0)
	Divorced	5 (2.3)
Educational attainment	Less than secondary	87 (39.5)
	Secondary school	119 (54.1)
	College	14 (6.4)
Employment status	Full time employment	96 (43.6)
	Part-time/Unemployed	124 (56.4)
Time spent in Malaysia (Months)	<12	14 (6.4)
	12–23	9 (4.1)
	24–36	10 (4.5)
	>36	187 (85.0)
Homelessness	Yes	1 (0.5)
	No	219 (99.5)
Separation from a family member	Yes	160 (72.7)
	No	60 (27.3)
Exposure to violence	Non-Exposed	125 (56.8)
	Exposed	95 (43.2)
Physical injuries (non-accidental)	Yes	63 (28.6)
	No	157 (71.4)
Perceived social support	Low/moderate	91 (41.4)
	High	129 (58.6)
Religious orientation * Mean (SD)	Overall ^a^	51.8 (8.0)
	Intrinsic ^b^	32.8 (4.4)
	Extrinsic-Personal ^c^	13.6 (2.5)
	Extrinsic-Social ^c^	5.4 (3.6)
Food security	Food secure	64 (29.1)
	Food insecure	156 (70.9)

Note. * For religious orientation higher mean score indicates higher religious orientation; ^a^ score range 14–70; ^b^ score range 8–40; and ^c^ score range 3–15.

**Table 2:** Prevalence of mental health disorders (MHDs) among participants (*n* = 220).

MHDs		Frequency *n* (%)	95% CI
MDD	Yes	71 (32.3)	0.26, 0.39
	No	149 (67.7)	-
GAD	Yes	92 (41.8)	0.36, 0.48
	No	128 (58.2)	-
PTSD	Yes	84 (38.2)	0.32, 0.45
	No	136 (61.8)	-

Note. MHDs, mental health disorders.

**Table 3:** Univariate and multivariate logistic regression for variables associated with major depressive disorder (MDD).

Variable	Univariate Logistic Regression		Multivariate Logistic Regression	
	Crude OR (95% CI)	*p*-Value	Adjusted OR (95%CI)	*p*-Value
Gender				
Male	1 (Reference)		1 (Reference)	
Female	1.58 (0.89, 2.78)	0.117 *	2.54 (1.21, 5.34)	0.014 **
Age (years)				
18–35	1 (Reference)			
36–55	0.92 (0.47, 1.83)	0.819	-	-
≥56	0.78 (0.26, 2,30)	0.649	-	-
Marital status				
Single	1 (Reference)			
Married	0.75 (0.36, 1.55)	0.437	-	-
Divorced	288 (0.00, 0.00)	0.999	-	-
Education				
Less than secondary	1 (Reference)		1 (Reference)	
Secondary school	0.68 (0.38, 1.23)	0.200 *	0.89 (0.45, 1.75)	0.726
College	0.45 (0.12, 1.72)	0.241 *	0.68 (0.15, 3.23)	0.631
Employment status				
Full-time employment	1 (Reference)			
Part time/Unemployment	1.41 (0.79, 2.50)	0.248 *	-	-
Time spent in Malaysia (Months)				
<12	6.67 (2.00, 22.21)	0.002 *	6.95 (1.89, 25.57)	0.004 **
12–23	3.33 (0.86, 12.90)	0.081 *	3.66 (0.81, 16.54)	0.092
24–36	2.67 (0.74, 9.60)	0.133 *	2.77 (0.61, 12.54)	0.185
>36	1 (Reference)		1 (Reference)	
Homelessness				
Yes	0.00 (0.00, 0.00)	1.000	-	-
No	1 (Reference)			
Separation from a family member				
Yes	1.16 (0.61, 2.20)	0.659	-	-
No	1 (Reference)			
Exposure to violence				
Non-Exposed	1 (Reference)			
Exposed	1.44 (0.82, 2.55)	0.207 *	-	-
Physical injuries			-	-
Yes	2.83 (1.54, 5.22)	0.001 *	3.25 (1.52, 6.96)	0.002 **
No	1 (Reference)		1 (Reference)	
Perceived social support				
Low/Moderate	2.71 (1.51, 4.84)	0.001 *	2.17 (1.13, 4.19)	0.021 **
High	1 (Reference)		1 (Reference)	
Religious orientation (Overall)	0.99 (0.96, 1.03)	0.653	-	-
Food security				
Food secure	1 (Reference)		1 (Reference)	
Food insecure	3.47 (1.64, 7.32)	0.001 *	2.77 (1.19, 6.47)	0.019 **

Note. OR, odds ratio; CI, confidence interval; the variable employment status and exposure to violence were excluded from multivariate analysis due to the presence of multicollinearity; * variables significant at 0.25 from the univariate analysis are entered into the multivariate analysis; and ** significant set at *p*-value < 0.05 for the multivariate analysis. The above analysis was adjusted for demographic variables. Hosmer–Lemeshow goodness-of-fit test chi-square = 3.836 (df = 7), *p* = 0.798. (Using enter method); overall religious orientation score was used to avoid multicollinearity between different dimensions of religious orientation.

**Table 4:** Univariate and multivariate logistic regression for variables associated with generalized anxiety disorder (GAD).

Variable	Univariate Logistic Regression		Multivariate Logistic Regression	
	Crude OR (95% CI)	*p*-Value	Adjusted OR (95%CI)	*p*-Value
Gender				
Male	1 (Reference)			
Female	1.21 (0.71, 2.06)	0.492	-	-
Age (years)				
18–35	1 (Reference)			
36–55	1.39 (0.73, 2.63)	0.317	-	-
≥56	1.56 (0.59, 4.16)	0.374	-	-
Marital status				
Single	1 (Reference)		1 (Reference)	
Married	0.88 (0.43, 1.76)	0.709	0.99 (0.47, 2.09)	0.975
Divorced	5.18 (0.53, 50.65)	0.158 *	4.21 (0.41, 43.16)	0.226
Education				
Less than secondary	1 (Reference)		1 (Reference)	
Secondary school	0.97 (0.55, 1.70)	0.906	0.97 (0.54, 1.75)	0.911
College	0.35 (0.09, 1.35)	0.128 *	0.31 (0.08, 1.24)	0.097
Employment status				
Full-time employment	1 (Reference)			
Part time/Unemployment	1.09 (0.64, 1.87)	0.752	-	-
Time spent in Malaysia (Months)				
<12	2.04 (0.68, 6.11)	0.205 *	2.14 (0.70, 6.56)	0.185
12–23	1.91 (0.50, 7.34)	0.347	1.67 (0.42, 6.73)	0.468
24–36	1.53 (0.43, 5.46)	0.515	1.47 (0.38, 5.67)	0.580
>36	1 (Reference)		1 (Reference)	
Homelessness				
Yes	1 (Reference)	1.000		
No	0.00 (0.00, 0.00)		-	-
Separation from a family member				
Yes	0.92 (0.50, 1.67)	0.780	-	-
No	1 (Reference)			
Exposure to violence				
Non-Exposed	1 (Reference)			
Exposed	1.02 (0.60, 1.75)	0.940	-	-
Physical injuries				
Yes	1.67 (0.93, 3.01)	0.089 *	1.75 (0.94, 3.25)	0.077
No	1 (Reference)		1 (Reference)	
Perceived social support				
Low/Moderate	1.00 (0.58, 1.72)	0.988	-	-
High	1 (Reference)			
Religious orientation (Overall)	0.98 (0.95, 1.02)	0.268	-	-
Food security				
Food secure	1 (Reference)			
Food insecure	1.29 (0.71, 2.34)	0.406	-	-

Note. OR, odds ratio; CI, confidence interval; * variables significant at 0.25 from the univariate analysis are entered into the multivariate analysis; and ** significant set at *p*-value < 0.05 for the multivariate analysis. The above analysis was adjusted for demographic variables. Hosmer–Lemeshow goodness-of-fit test chi-square = 2.080 (df = 6), *p* = 0.912. (Using enter method). The overall religious orientation score was used to avoid multicollinearity between different dimensions of religious orientation.

**Table 5:** Univariate and multivariate logistic regression for variables associated with post-traumatic stress disorder (PTSD).

Variable	Univariate Logistic Regression		Multivariate Logistic Regression	
	Crude OR (95% CI)	*p*-Value	Adjusted OR (95%CI)	*p*-Value
Gender				
Male	1 (Reference)		1 (Reference)	
Female	0.59 (0.34, 1.03)	0.063 *	0.60 (0.27, 1.35)	0.217
Age (years)				
18–35	1 (Reference)		1 (Reference)	
36–55	1.18 (0.62, 2.25)	0.609	1.16 (0.45, 2.96)	0.762
≥56	0.31 (0.09, 1.12)	0.075 *	0.36 (0.07, 1.95)	0.237
Marital status				
Single	1 (Reference)			
Married	1.26 (0.61, 2.62)	0.537	-	-
Divorced	3.00 (0.45, 20.24)	0.259	-	-
Education				
Less than secondary	1 (Reference)			
Secondary school	0.96 (0.54, 1.70)	0.889	-	-
College	1.64 (0.53, 5.08)	0.395	-	-
Employment status				
Full-time employment	1 (Reference)			
Part time/Unemployment	0.97 (0.56, 1.68)	0.923	-	-
Time spent in Malaysia (Months)				
<12	0.85 (0.27, 2.63)	0.776	-	-
12–23	0.44 (0.09, 2.16)	0.309	-	-
24–36	0.65 (0.16, 2.61)	0.548	-	-
>36	1 (Reference)			
Homelessness				
Yes	1 (Reference)	1.000		
No	0.00 (0.00, 0.00)		-	-
Separation from a family member				
Yes	1.82 (0.96, 3.47)	0.068 *		
No	1 (Reference)		-	-
Exposure to violence				
Non-Exposed	1 (Reference)		1 (Reference)	
Exposed	34.39 (15.75, 75.10)	0.000 *	38.46 (16.27, 90.91)	0.000 **
Physical injuries				
Yes	21.54 (9.86, 47.07)	0.000 *		
No	1 (Reference)		-	-
Perceived social support				
Low/Moderate	0.94 (0.54, 1.64)	0.834	-	- -
High	1 (Reference)			
Religious orientation (Overall)	1.03 (0.99, 1.06)	0.154 *	1.05 (0.99, 1.10)	0.069
Food security				
Food secure	1 (Reference)		1 (Reference)	-
Food insecure	3.71 (1.84, 7.50)	0.000 *	3.74 (1.41, 9.91)	0.008 **

Note. OR, odds ratio; CI, confidence interval; the variable separation from a family member and physical injuries were excluded from multivariate analysis due to presence of multicollinearity; * variables significant at 0.25 from the univariate analysis are entered into the multivariate analysis; and ** significant set at *p*-value < 0.05 for the multivariate analysis. The above analysis was adjusted for demographic variables. Hosmer–Lemeshow goodness-of-fit test chi-square = 9.503 (df = 8), *p* = 0.302. (using the enter method). The overall religious orientation score was used to avoid multicollinearity between different dimensions of religious orientation.

**Figure A1:**
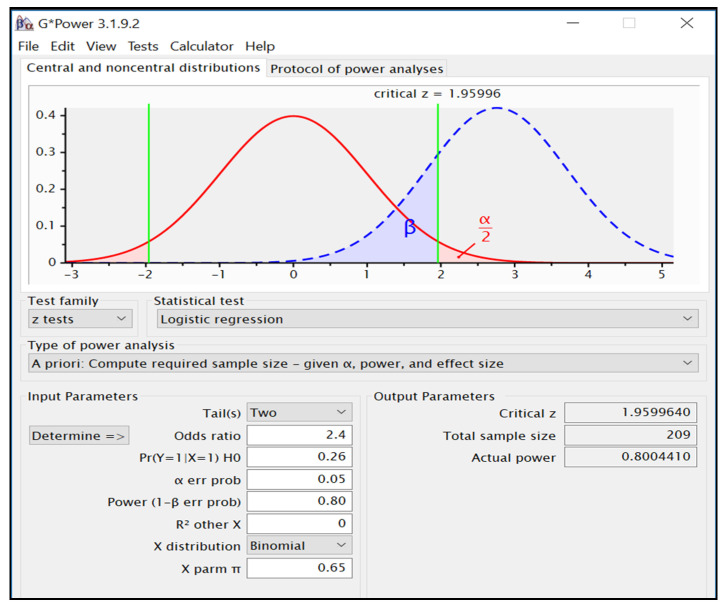
G* power sample size output.

## Appendix B. Test of Multicollinearity

**Table A1:** Testing for multicollinearity among the independent variables significant at 0.25 in the univariate analysis examining the factors associated with MDD.

	Gender	Time in Malaysia	Physically Injured	Social Support	Food Security	Exposure to Violence	Employment	Education
Gender		0.144	0.298	0.056	0.135	0.090	0.631	0.270
Time in Malaysia	0.144		0.030	0.116	0.069	0.182	0.177	0.103
Physically injured	0.298	0.030		0.162	0.295	0.463	0.132	0.126
Social Support	0.056	0.116	0.162		0.192	0.043	0.013	0.154
Food Security	0.135	0.069	0.295	0.192		0.195	0.120	0.123
Exposure to violence	0.090	0.182	0.463	0.043	0.195		0.010	0.036
Employment	0.631	0.177	0.132	0.013	0.120	0.010		0.169
Education	0.270	0.103	0.126	0.154	0.123	0.036	0.169	

Note. Cramer’s V values of more than 0.3 indicate evidence of multicollinearity among variables.

**Table A2:** Testing for multicollinearity among the independent variables significant at 0.25 in the univariate analysis examining the factors associated with GAD.

	Marital Status	Time in Malaysia	Physically Injured	Education
Marital status		0.153	0.172	0.149
Time in Malaysia	0.153		0.030	0.103
Physically injured	0.172	0.030		0.126
Education	0.149	0.103	0.126	

Note. Cramer’s V values of more than 0.3 indicate evidence of multicollinearity among variables.

**Table A3:** Testing for multicollinearity among the independent variables significant at 0.25 in the univariate analysis examining the factors associated with PTSD.

	Gender	Age (Years)	Separation from Family Members	Exposure to Violence	Physical Injuries	Food Security
Gender		0.125	0.401	0.090	0.298	0.135
Age (years)	0.125		0.246	0.106	0.129	0.090
Separation from family members	0.401	0.246		0.122	0.298	0.033
Exposure to violence	0.090	0.106	0.122		0.463	0.195
Physical injuries	0.298	0.129	0.298	0.463		0.295
Food security	0.135	0.090	0.033	0.195	0.295	

Note. Cramer’s V values of more than 0.3 indicate evidence of multicollinearity among variables.
